# Prevalence and risk factors of active tuberculosis in patients with rheumatic diseases: a multi-center, cross-sectional study in China

**DOI:** 10.1080/22221751.2021.2004864

**Published:** 2021-12-06

**Authors:** Xiaoqing Liu, Lifan Zhang, Fengchun Zhang, Xiaofeng Zeng, Yan Zhao, Qian Wang, Shengyun Liu, Xiaoxia Zuo, Zhiyi Zhang, Huaxiang Wu, Sheng Chen, Hongbin Li, Ping Zhu, Lijun Wu, Wencheng Qi, Yi Liu, Miaojia Zhang, Huaxiang Liu, Dong Xu, Wenjie Zheng, Yueqiu Zhang, Xiaochun Shi, Lishuai Han, Yaou Zhou, Yanping Zhao, Wenwen Wang, Ting Li, Ning Tie, Kui Zhang, Cainan Luo, Baoqi Gong, Yi Zhao, Chengyin Lv, Lijun Song, Qingjun Wu, Yunyun Fei, Lei Zhang, Hui Luo, Jiaying Sun, Jing Xue, Liyang Gu, Jing Wang, Qing Han, Kuerbanjiang Yimaity, Jiaxin Zhou, Lidan Zhao, Sainan Bian, Wufang Qi, Yanhong Li, Yujing Zhu, Huijun Han, Susu Liao, Gaifen Liu

**Affiliations:** a Division of Infectious Diseases, Department of Internal Medicine, State Key Laboratory of Complex Severe and Rare Disease, Peking Union Medical College Hospital, Chinese Academy of Medical Sciences & Peking Union Medical College, Beijing, People’s Republic of China; b Centre for Tuberculosis Research, Chinese Academy of Medical Sciences & Peking Union Medical College, Beijing, People’s Republic of China; c Clinical Epidemiology Unit, International Epidemiology Network, Peking Union Medical College Hospital, Chinese Academy of Medical Science, Beijing, People’s Republic of China; d Department of Rheumatology and Clinical Immunology, Peking Union Medical College Hospital, Chinese Academy of Medical Sciences & Peking Union Medical College, Key Laboratory of Rheumatology & Clinical Immunology, Ministry of Education, Beijing, People’s Republic of China; e Department of Rheumatology and Immunology, The First Affiliated Hospital of Zhengzhou University, Zhengzhou, People’s Republic of China; f Department of Rheumatology and Immunology, Xiangya Hospital, Central South University, Changsha, People’s Republic of China; g Department of Rheumatology and Immunology, The First Affiliated Hospital of Harbin Medical University, Harbin, People’s Republic of China; h Department of Rheumatology, The Second Affiliated Hospital of Zhejiang University School of Medicine, Hangzhou, People’s Republic of China; i Department of Rheumatology, Renji Hospital, School of Medicine, Shanghai Jiao Tong University, Shanghai, People’s Republic of China; j Department of Rheumatology and Immunology, The Affiliated Hospital of Inner Mongolia Medical University, Hohhot, People’s Republic of China; k Department of Clinical Immunology, Xijing Hospital, Fourth Military Medical University, Xi'an, People’s Republic of China; l Department of Rheumatology, People's Hospital of Xinjiang Uygur Autonomous Region, Urumchi, People’s Republic of China; m Department of Rheumatology, Tianjin First Central Hospital, Tianjin, People’s Republic of China; n Department of Rheumatology and Immunology, West China Hospital, Sichuan University, Chengdu, People’s Republic of China; o Department of Rheumatology, The first affiliated hospital of Nanjing Medical University, Nanjing, People’s Republic of China; p Department of Rheumatology, Qilu Hospital of Shandong University, Ji'nan, People’s Republic of China; q Department of Epidemiology and Biostatistics, Institute of Basic Medical Sciences, Chinese Academy of Medical Sciences & School of Basic Medicine, Peking Union Medical College, Beijing, People’s Republic of China; r China National Clinical Research Center for Neurological Diseases, Department of Neurology, Beijing Tiantan Hospital, Capital Medical University, Beijing, People’s Republic of China

**Keywords:** Active tuberculosis, rheumatic disease, prevalence, risk factors, China

## Abstract

Evidence of active tuberculosis (ATB) in patients with rheumatic diseases are research priorities but limited data from China have been reported. Research targeting patients not taking anti-TNF biologics are especially insufficient. We aimed to investigate the prevalence and risk factors of ATB in this at-risk population. We conducted a tertiary hospital-based, multi-center, cross-sectional study by using stratified multi-stage cluster sampling strategy to screen ATB in patients with rheumatic diseases. We estimated the prevalence of ATB in patients with rheumatic diseases and identified risk factors among those who were not taking anti-TNF biologic. A total of 13,550 eligible patients were enrolled, and the result showed the standardized prevalence of ATB according to the composition ratio of various types of rheumatic disease was 882/100000 (95% confidence interval (CI): 706-1057). Multivariable logistic regression analysis in patients not taking anti-TNF biologics showed that the independent risk factors of ATB were having systemic lupus erythematosus (SLE) (OR=2.722, 95% CI: 1.437-5.159, *p*=0.002), having Behcet’s disease (BD) (OR= 5.261, 95% CI: 2.071-13.365, *p*<0.001), taking azathioprine(AZA) within the past two years (OR=2.095, 95% CI: 0.986-4.450, *p*=0.054), exposing to glucocorticoids ≥30mg/d for more than four weeks within the past two years (OR=2.031, 95% CI: 1.247-3.309, *p*=0.004) and having evidences of previous TB (OR= 6.185, 95% CI: 3.487-10.969, *p*<0.001). The prevalence of ATB was higher in patients with rheumatic diseases compared to the general population. Patients with SLE or BD, prolonged exposure to moderate to high dose of glucocorticoids and previous TB were independent risk factors for ATB.

## Background

China has the third largest number of tuberculosis (TB) cases in the world [1]. The prevalence of active pulmonary TB was 459/100,000 in population aged over 15 in 2010 [2]. In 2019, 833,000 people were newly diagnosed with TB in China, accounting for nearly 8.4% of all TB cases worldwide [1]. Rheumatic disease is also a serious problem in China. It was estimated that China has approximately 20 million patients with rheumatic disease [3,4]. Due to the primary disease as well as the use of immunosuppressants, patients with rheumatic disease were at high risk for developing ATB.

The coexistence of TB and rheumatic diseases requires many complex considerations. TB infection can make the diagnosis and treatment of rheumatic diseases complicated. On the other hand, rheumatic patients are usually under immunocompromised conditions, leading to prolonged infection duration and higher mortality rate [5,6]. Therefore, prevention of new infections of *Mycobacterium tuberculosis* (MTB) and the progression to ATB is especially crucial for patients with rheumatic diseases.

WHO strongly recommends that patients initiating anti-TNF treatment should be systematically tested and treated for latent TB infection (LTBI). However, there is no recommendation of TB preventive treatment for patients with rheumatic conditions and receiving steroid treatment [7–9]. In China, the expert consensus on treatment for LTBI was reached for rheumatic patients receiving TNF antagonists in 2014, but no recommendation of TB prevention has been made when anti-TNF drugs are not administered until now [10].

Tuberculosis prevention and control in patients with rheumatic diseases faces two difficulties. Firstly, the prevalence of ATB in this population is unknown. This is the first step for TB control, but national epidemiological data is still lacking. Secondly, the risk factors of ATB in rheumatic patients not receiving anti-TNF treatment are unclear. Given the medical resource constraint and the fact that anti-TB therapy may induce severe adverse drug reactions when combined with rheumatic diseases [11], TB preventive treatment for all rheumatic patients may not be an optimal option. Identifying risk factors offer doctors reliable information in assessing the risk of ATB in patients with rheumatic diseases, which can greatly facilitate the generation of efficient prevention strategies based on clinical individualized risk assessment. Previous case–control and cohort studies have shown that the use of glucocorticoids and some immunosuppressants can increase the risk of ATB in patients with rheumatic disorders [12–16]. However, the findings are inconsistent, and most of the data come from countries or regions with low or medium TB burden.

In China, due to the complexity of the diagnosis and treatment of rheumatic diseases, only tertiary general hospitals are required by the government to set up departments of rheumatology. Under this circumstance, a multi-center, cross-sectional study (ETHERTB) based on tertiary general hospitals was conducted to investigate the prevalence of ATB among patients with rheumatic diseases and to determine risk factors associated with ATB in patients not taking anti-TNF medications.

## Methods

### Study design and population

A tertiary hospital-based, multi-center, cross-sectional study was conducted. A consecutive sample of eligible outpatients and inpatients with rheumatic diseases from 13 tertiary general hospitals was recruited and screened for ATB between September 16, 2014 and March 15, 2016.

Inclusion criteria were as follows: 1) aged over 15 years; and 2) satisfied the classification criteria of rheumatic diseases, including systemic lupus erythematosus (SLE), rheumatoid arthritis (RA), Sjogren's syndrome (SS), systemic sclerosis (SSc), mixed connective tissue disease (MCTD), polymyositis (PM), Takayasu arthritis (TA), giant cell arthritis (GCA), polyarteritis nodosa (PAN), granulomatosis with polyangiitis (GPA), microscopic polyarteritis (MPA), eosinophilic granulomatosis with polyangiitis (EGPA), Behcet’s disease (BD), ankylosing spondylitis (AS) and psoriatic arthritis (PsA). Pregnant women were excluded. Eligibility of patients were independently reviewed and verified by two rheumatologists. Disagreement was resolved by consulting a third rheumatologist.

This study was approved by the Ethics Committees of Peking Union Medical College Hospital (No.S-715) and 12 participating hospitals. Written informed consents were obtained from all patients and their legal guardians if necessary.

### Sampling

TB epidemic is closely related to local economic condition. The regional economic disparity in China leads to the distinct burden of TB among different regions [2]. Therefore, we adopted a stratified multi-stage cluster sampling strategy in order to recruit a representative sample of patients for this study. Firstly, China was divided into three regions based on geographic location and economic status, namely, eastern, central, and western areas. Then, we used simple random sampling method to select the province in each region. We estimated that there was no significant difference in the prevalence of rheumatic diseases among different regions [4,17], the number of cases with rheumatic diseases would be proportional to the total population. So, we determined the number of provinces according to the population size of different regions and selected 6 out of 9, 3 out of 10 and 4 out of 12 provinces, municipalities or autonomous regions were selected from the eastern, central, and western region, respectively. Secondly, for each selected province, municipality, or autonomous region, one tertiary general hospital was randomly chosen as study site (Supplementary Figure 1). Finally, the required sample size for each region was equally allocated to the sites in that region and eligible outpatients and inpatients with rheumatic diseases were consecutively screened and enrolled.

Firstly, the required sample size of each region was calculated using the formula of $n=\frac{Z^{2}\cdot P\cdot (1-P)}{d^{2}}$ where n is the sample size, Z is the statistic corresponding to 95% confidence interval, P is expected prevalence of ATB in patients with rheumatic diseases and d is precision. We assumed there was no significant difference in the prevalence of rhematic disease among different regions [4,17] and the prevalence of ATB in rheumatic patients was a 10-fold of prevalence of ATB in the general population. So we set the expected prevalence of ATB in rheumatic patients to be 3%, 5% and 7% in the eastern, central and western regions, respectively [2]. And d was set as one-fifth of the prevalence. Confirming the diagnosis can sometimes be complicated and may require regular follow-up. Then, the sample size was amplified by considering the loss-to-follow-up rate and design effect. We assumed approximately 10% loss to follow-up. Also, we set the design effect as 2 by considering the stratified multi-stage cluster sampling method. Accordingly, the sample sizes for the eastern, central and western regions were estimated to be 6900, 4056, and 2836, respectively.

### Screening for ATB

**Figure 1.**
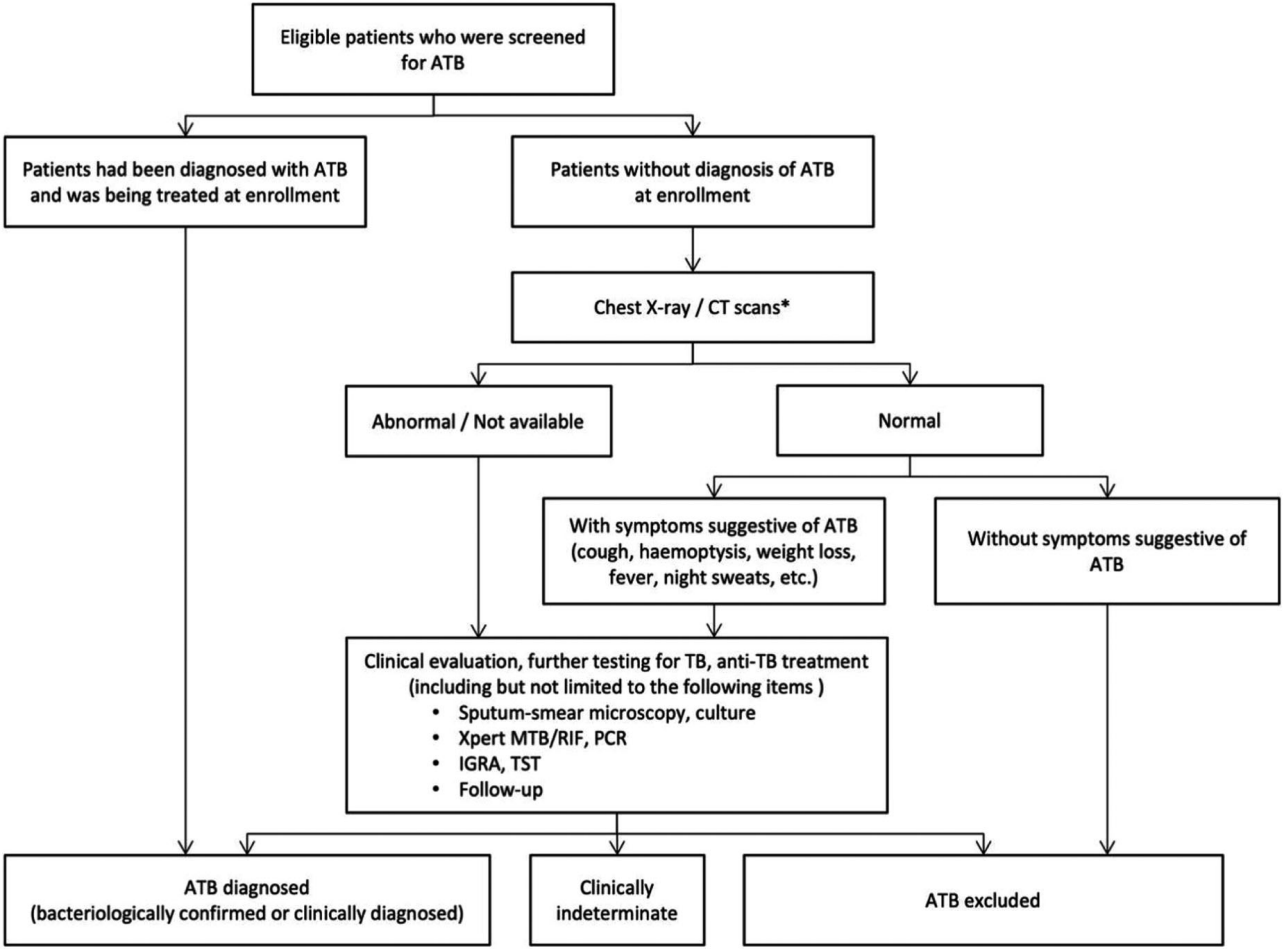
Screening process for ATB in enrolled patients with rheumatic diseases. ATB=active tuberculosis, IGRA=interferon-gamma release assay, TST=tuberculin skin test. *Whether to choose Chest X-ray or CT scans was decided by the clinicians based on each patient's clinical condition.

Chest X-rays or CT scans were performed on all patients for ATB screening. When ATB was suspected, specimens from lesions including sputum, other respiratory secretions, cerebrospinal fluid, pleural effusion, ascites and pericardial fluid were collected for acid-fast staining and TB culture. Histopathological examinations were conducted when necessary. The screening algorithm of ATB is summarized in Figure 1.

The diagnosis of ATB was independently made by two respiratory or infectious disease specialists. Disagreement was resolved by consulting a third senior physician. The diagnostic criteria of ATB are listed in Table 1.

**Table 1.** Categorization of the study population.

Diagnostic Category	Criteria
Microbiologically /histologically confirmed TB	A biological specimen was positive by smear microscope, culture or Xpert MTB/RIF and (or) histological changes in lesions were typical, such as caseous necrosis, epithelioid granulomatous and etc.
Clinically diagnosed TB	Patients had characteristic symptoms, including fever, cough, chest pain, night sweats, weight loss and etc. Laboratory and imaging findings were highly suggestive for MTB and diagnostic anti-TB treatment was effective.
Clinically indeterminate	A final diagnosis of tuberculosis was neither confirmed nor reliably excluded
ATB excluded	Bacteriological or histological examinations showed no evidence for ATB. If the patient was originally suspected of TB, an alternative diagnosis should be identified and eventually confirmed by diagnostic therapy.

### Potential risk factors for ATB

Age, gender, type of rheumatic disease, duration of rheumatic disease, dosage and duration of glucocorticoids within the past two years, use of immunosuppressants within the past two years and evidence of previous TB were collected as variables for the risk factor analysis.

### Statistical analysis

Normality of the numerical variables were tested by the Kolmogorov–Smirnov test. Numerical variables with normal distributions were expressed as mean ± standard deviation (SD), non-normal variables were described as median and interquartile range [IQR]. Categorical variables were presented as frequencies and proportions. Comparisons of continuous variables between patients with and without ATB were performed using t-test and Mann–Whitney U test for normal and non-normal data respectively. Categorical data were compared using Chi-squared or Fisher's exact test as appropriate.

The prevalence of ATB in each rheumatic disease was calculated and a crude overall prevalence was estimated as well. The risk of ATB varied among different rheumatic diseases. In order to avoid bias due to differences in the disease composition between the included patients and the general rheumatic patients, we collected the disease type data of all patients with rheumatic disease in 13 sub-centers throughout 2014 and standardized the overall ATB prevalence according to the weight of the disease types. We estimated the prevalence of ATB based on Possion distribution. 95% confidence interval (CI) of the prevalence was presented.

We used univariate analysis and multivariable logistic regressions to identify the risk factors of ATB. Patients who were on anti-TNF biologics and TB preventive treatment were excluded from the analysis. Age, gender, the type of rheumatic disease, the duration of rheumatic disease, use of immunosuppressants within past two years, dosage and duration of glucocorticoids within the past two years and evidence of previous TB were included in the univariate analysis. Variables that were considered clinically relevant or that showed a significant association with ATB in the univariate analysis were entered into the multivariable logistic regressions model. Given the number of events available, variables included in the multivariate logistic model were carefully chosen to ensure parsimony of the final model (Backward LR, entry 0.05, removal 0.10).

All statistical analyses were performed with SPSS (IBM SPSS Statistics for Windows, Version 22.0. Armonk, NY: IBM Corp). A *p* < 0.05 was considered statistically significant.

## Results

**Figure 2.**
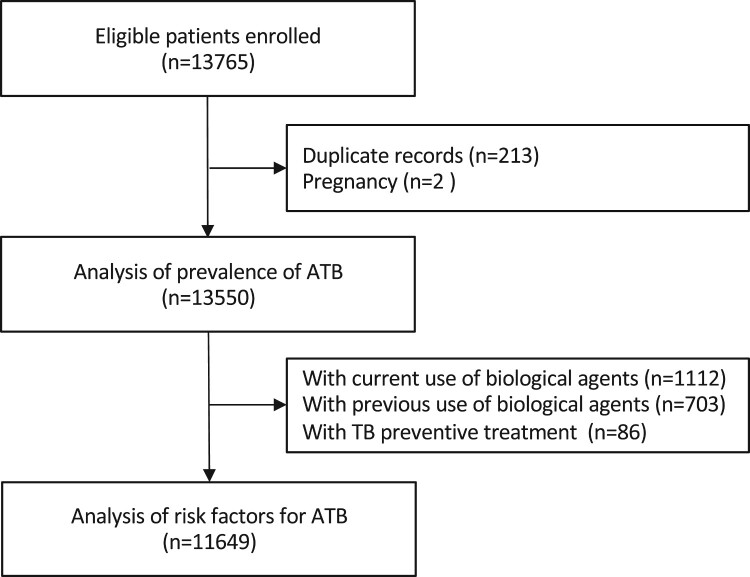
Flowchart of the study. ATB=active tuberculosis.

A total of 13,550 patients were finally included for the estimation of ATB prevalence. After excluding participants with history of anti-TNF biologics or TB preventive treatment, 11,649 rheumatic patients were included in the analysis for identifying risk factors (Figure 2). The median age of 13,550 patients was 45 [IQR 33-56] years old and 10,286 patients (75.9%) were female. Patients with SLE (2959, 21.8%) and RA (5116, 37.8%) had the largest population, accounting for nearly 60%. General characteristics of participants are shown in Table 2.

**Table 2.** General characteristics of 13550 enrolled patients with rheumatic conditions.

	ATB	Non-ATB
	Number of patients/Total number (%)	Number of patients/Total number (%)
Male	30/105 (28.6)	3234/13445 (24.1)
Median age [IQR], year	42 [28–53]	45 [33–56]
Education level		
College and above	28/83 (33.7)	2939/11081 (26.5)
Senior high school	15/83 (18.1)	2549/11081 (23.0)
Junior high school	25/83 (30.1)	3372/11081 (30.4)
Primary school	13/83 (15.7)	1696/11081 (15.3)
Illiterate	2/83 (2.4)	525/11081 (4.7)
Type of rheumatic diseases		
RA	19/105 (18.1)	5097/13445 (37.9)
SLE	41/105 (39.0)	2918/13445 (21.7)
SS	9/105 (8.6)	1271/13445 (9.5)
SSc	5/105 (4.8)	472/13445 (3.5)
MCTD	0/105 (0)	329/13445 (2.4)
PM	1/105 (1.0)	220/13445 (1.6)
DM	2/105 (1.9)	521/13445 (3.9)
TA	4/105 (3.8)	156/13445 (1.2)
BD	7/105 (6.7)	304/13445 (2.3)
Other systemic vasculitis†	2/105 (1.9)	206/13445 (1.5)
AS	15(/105 (14.3)	1173/13445 (12.9)
PsA	0/105 (0)	217/13445 (1.6)
Median duration of rheumatic diseases [IQR], month	24 [7–72]	14 [1–50]
Medications within the past two years		
GCs	79/103 (76.7)	8768/13189 (66.5)
Immunosuppressants	70/103 (68.0)	8770/13235 (66.3)
Biological agents	12/101 (11.7)	1834/13195 (13.9)
Complications		
Diabetes Mellitus	7/105 (6.7)	528/13226 (4.0)
Malignancy	0/104 (0)	66/13246 (0.5)
Chronic renal failure	1/104 (1.0)	88/13346 (0.7)
Silicosis	0/103 (0)	13/13338 (0.1)
Exposure to TB	1/86 (1.2)	160/13307 (1.2)
With evidence of previous TB	18/98 (18.4)	458/13381 (3.4)

SLE=systemic lupus erythematosus. RA=rheumatoid arthritis. SS=Sjogren’s syndrome. SSc=systemic sclerosis. MCTD=mixed connective tissue diseases. PM=polymyositis. DM=dermatomyositis. TA=Takayasu's arteritis. BD=Behcet’s disease. SpA=spondyloarthropathy. GCs=glucocorticoids.

†Including giant cell arteritis (GCA), polyarteritis nodosa (PAN), granulomatosis with polyangiitis (GPA), microscopic polyangiitis (MPA) and eosinophilic granulomatosis with polyangiitis (EGPA).

### Prevalence of ATB

Among 13,550 patients with rheumatic diseases, 105 cases were diagnosed with ATB, of which 32 (30.5%) cases were confirmed microbiologically, 2 (1.9%) cases were diagnosed histologically, and 71 (67.6%) cases were diagnosed clinically. The crude estimation of overall ATB prevalence was 775 (95% CI: 634-937) per 100,000. After being adjusted for disease composition, the standardized prevalence was 882 (95% CI: 706-1057) per 100,000 (Table 3). The prevalence of ATB is varied across different types of rheumatic disease, ranging from 371/100,000–2500/100,000.

**Table 3.** Prevalence of ATB among patients with rheumatic diseases.

Type of rheumatic diseases	Number of patients with ATB	Total number of patients	Prevalence of ATB (/100,000 population) (95% CI)
SLE	41	2959	1386 (964-1807)
RA	19	5116	371 (205-538)
SS	9	1280	703(245-1161)
SSc	5	477	1048(131-1966)
MCTD	0	329	/
PM	1	221	453(0- 1344)
DM	2	523	382 (0-913)
TA	4	160	2500(55- 4945)
BD	7	311	2251(593- 3908)
Other systemic vasculitis*	2	208	962(0-2299)
AS	15	1749	858(425-1290)
PsA	0	217	/
Overall prevalence	105	13550	775(634-937)
Standardized overall prevalence	105	13550	882(706-1057)

SLE=systemic lupus erythematosus. RA=rheumatoid arthritis. SS=Sjogren’s syndrome. SSc=systemic sclerosis. MCTD=mixed connective tissue diseases. PM=polymyositis. DM=dermatomyositis. TA=Takayasu's arteritis. BD=Behcet’s disease. SpA=spondyloarthropathy.

*Including giant cell arteritis (GCA), polyarteritis nodosa (PAN), granulomatosis with polyangiitis (GPA), microscopic polyangiitis (MPA) and eosinophilic granulomatosis with polyangiitis (EGPA).

Among the 32 patients with microbiologic diagnosis, 26 (81.3%) were positive for acid-fast staining, 7 (21.9%) were positive for TB culture, and 7 (21.9%) were positive for MTB nucleic acid detection. According to the latest Chinese standard of TB classification (WS196-2017) [18], 88 (83.8%) cases had pulmonary TB, including 74 (84.1%) primary or secondary pulmonary TB, 10 (11.4%) TB pleurisy, 3 (3.4%) Miliary TB and 1 (1.1%) endobronchial TB (Table 4). Involvement of multiple organs was found in 15 (14.3%) patients. 48.8% (20/41) SLE patients and 57.1% (4/7) BD patients had extra-pulmonary TB lesions, which was significantly higher than patients with other rheumatic conditions (24/48 vs. 15/57, *p*=0.012). Notable infected sites include lung (77 cases, 62.1%), pleura (13 cases, 10.5%), lymph nodes (8 cases, 6.5%), bones (8 cases, 6.5%), meninges (6 cases, 4.8%), skin (3 cases, 2.4%), urinary system (2 cases, 1.6%), and liver (2 cases, 1.6%).

**Table 4.** Classification of ATB patients with rheumatic diseases (WS196-2017).

Tuberculosis classification	N (%) (n=105)
Pulmonary TB	88 (83.8)
Lung	62 (59.0)
Lung + lymph nodes	3 (2.9)
Lung + Pleura	2 (1.9)
Lung + meninges	2 (1.9)
Lung + bone	1 (1.0)
Lung + skin	1 (1.0)
Lung + skin+ bone	1 (1.0)
Lung + Pleura + pericardium	1 (1.0)
Lung + meninges + liver + spleen	1 (1.0)
Pleura	10 (9.5)
Miliary TB	2 (1.9)
Miliary TB + meninges	1 (1.0)
Bronchia	1 (1.0)
Extra-pulmonary TB	17 (16.2)
Lymph nodes	5 (4.8)
Bone	5 (4.8)
Urinary system	2 (1.9)
Meninges	1 (1.0)
Skin	1 (1.0)
Uvea	1 (1.0)
Bone + meninges	1 (1.0)
Liver + spleen	1 (1.0)

### Risk factors for ATB

Risk factors for ATB were analyzed in 11,649 subjects who had no history of anti-TNF biologics or TB preventive treatment. Eighty-four of these patients were diagnosed with ATB. The results of the univariate analysis are shown in Table 5. Variables including age, type of rheumatic disease, use of CTX/MMF/AZA/LEF within past two years, dosage and duration of glucocorticoids within past two years, and with evidence of previous TB were entered into the multivariable logistic regressions model (Supplementary Material), and the results showed that having SLE (OR=2.722, 95% CI: 1.437-5.159, *p*=0.002), BD (OR= 5.261, 95% CI: 2.071-13.365, *p*<0.001), use of AZA within the past two years (OR=2.095, 95% CI: 0.986-4.450, *p*=0.054), exposure to glucocorticoids with dose ≥30mg/d for more than four weeks within the past two years (OR=2.031, 95% CI: 1.247-3.309, *p*=0.004) and history of previous TB (OR= 6.185, 95% CI: 3.487-10.969, *p*<0.001) were independent risk factors of ATB (Table 6).

**Table 5.** Potential risk factors of ATB in patients with rheumatic diseases in univariate analysis.

			Univariate analysis	
	ATB	Non-ATB	OR (95% CI)	*P* value
	n=84	n=11565		
Gender				
Female	62	9100	1	
Male	22	2465	1.310 (0.804-2.135)	0.279
Age(years) Median [IQR]	42 [29–54]	46 [34–56]	0.982 (0.966-0.997)	0.020
Type of rheumatic diseases				
RA	15	4238	1	
SLE	37	2783	**3.756 (2.058-6.857)**	**<0.001**
BD	7	276	**7.166 (2.898-17.720)**	**<0.001**
Other rheumatic diseases*	25	4268	1.655 (0.871-3.143)	0.124
Duration of rheumatic diseases (months), Median [IQR]	26 [7–74]	16 [1–59]	1.001 (0.998-1.004)	0.415
Use of CTX within past two years				
No	65	9953	1	
Yes	19	1612	**1.805 (1.080-3.017)**	**0.024**
Use of MMF within past two years				
No	72	10694	1	
Yes	12	871	**2.046 (1.106-3.785)**	**0.022**
Use of MTX within past two years				
No	66	8040	1	
Yes	18	3525	0.622 (0.369-1.049)	0.075
Use of AZA within past two years				
No	76	11191	1	
Yes	8	374	**3.150 (1.509-6.574)**	**0.002**
Use of LEF within past two years				
No	69	9137	1	
Yes	15	2428	0.818 (0.467-1.432)	0.482
Use of CsA within past two years				
No	81	11295	1	
Yes	3	270	1.549 (0.486-4.936)	0.459
Use of FK506 within past two years				
No	83	11388	1	
Yes	1	177	0.775 (0.107-5.599)	0.801
Duration of GCs≥30mg/d within past two years (weeks)				
0-4	56	9870	1	
>4	28	1695	**2.912 (1.844-4.596)**	**<0.001**
With evidence of previous TB				
No	69	11180	1	
Yes	15	385	**6.993 (3.943-12.403)**	**<0.001**

SLE=systemic lupus erythematosus. RA=rheumatoid arthritis. SS=Sjogren’s syndrome. SSc=systemic sclerosis. MCTD=mixed connective tissue diseases. PM=polymyositis. DM=dermatomyositis. TA=Takayasu's arteritis. BD=Behcet’s disease. SpA=spondyloarthropathy. CTX=Cyclophosphamide. MMF=Mycophenolate mofetil. MTX=Methotrexate. AZA=Azathioprine. LEF=Leflunomide. CsA=Cyclosporine A. FK506=Tacrolimus.

*Including Sjogren's syndrome (SS), systemic sclerosis (SSc), mixed connective tissue disease (MCTD), polymyositis (PM), dermatomyositis (DM), Takayasu arthritis (TA), giant cell arthritis (GCA), polyarteritis nodosa (PAN), granulomatosis with polyangiitis (GPA), microscopic polyarteritis (MPA), eosinophilic granulomatosis with polyangiitis (EGPA), ankylosing spondylitis (AS) and psoriatic arthritis (PsA).

**Table 6.** Potential risk factors of ATB in patients with rheumatic diseases in multivariable logistic regression.

			Multivariable analysis	
	ATB	Non-ATB	OR (95% CI)	*P* value
	n=84	n=11565		
Type of rheumatic diseases				
RA	15	4238	1	**<0.001**
SLE	37	2783	**2.722 (1.437-5.159)**	**0.002**
BD	7	276	**5.261 (2.071-13.365)**	**<0.001**
Other rheumatic diseases*	25	4268	1.320 (0.684-2.549)	0.408
Use of AZA within past two years				
No	76	11191	1	
Yes	8	374	2.095 (0.986-4.450)	0.054
Duration of GCs≥30mg/d within past two years (weeks)				
0-4	56	9870	**1**	
>4	28	1695	**2.031 (1.247-3.309)**	**0.004**
With evidence of previous TB				
No	69	11180	**1**	
Yes	15	385	**6.185 (3.487-10.969)**	**<0.001**

RA=rheumatoid arthritis. SLE=systemic lupus erythematosus. BD=Behcet’s disease. AZA=azathioprine. GCs=glucocorticoids.

*Including Sjogren's syndrome (SS), systemic sclerosis (SSc), mixed connective tissue disease (MCTD), polymyositis (PM), dermatomyositis (DM), Takayasu arthritis (TA), giant cell arthritis (GCA), polyarteritis nodosa (PAN), granulomatosis with polyangiitis (GPA), microscopic polyarteritis (MPA), eosinophilic granulomatosis with polyangiitis (EGPA), ankylosing spondylitis (AS) and psoriatic arthritis (PsA)

## Discussion

This is the first study conducted using stratified multi-stage cluster sampling design to estimate the prevalence of ATB and to identify risk factors in patients with rheumatic diseases in China. This will contribute to TB epidemiological data and provide information on TB risk stratification in this patient population, which supports the development of precise TB prevention and management strategies for this vulnerable population.

This study revealed that the standardized prevalence of ATB was 882/100,000 in patients with rheumatic diseases, nearly twice as much as the prevalence in the general population (459/100,000) [2]. Patients with different type of rheumatic diseases are varied in the risk of ATB. SLE and BD patients were most likely to have ATB with a prevalence of 3.0 and 4.9 times higher than the general population, respectively. Previous studies reported the prevalence of ATB in patients with SLE ranging from 1.3% to 5.3% [19–23], which were based on retrospective analysis of medical records rather than epidemiological survey. The prevalence of ATB in patients with SLE in Mexico (1.3%) and Colombia (1.4%) was similar to 1386/100,000 in our study. However, the prevalence from Asia (2.0% to 5.3%) was higher than that in this study, and these studies only included hospitalized patients with more severe conditions, which may result in an overestimation of the prevalence due to selection bias. Few studies reported the prevalence of ATB in patients with BD. Our previous study showed that 5.4% of the hospitalized patients with BD were diagnosed with ATB[24]. Similarly, the prevalence of ATB in hospitalized patients with BD was higher than that obtained in this epidemiological survey (2251/100,000). In this study, the prevalence of ATB in RA patients was similar to that in the general population, which may be related to the prophylaxis of tuberculosis in patients initiating anti-TNF treatment [25].

This study explored risk factors associated with ATB in rheumatic patients not taking anti-TNF medications. The independent risk factors for ATB included BD and SLE. BD itself may produce a defect in cell-mediated immunity, which increased the risk of developing ATB [26,27]. Additionally, TB infection may also be involved in inducing BD in individuals with genetic susceptibility [28]. According to a retrospective study from Singapore, SLE was an independent risk factor for ATB (OR = 4.6, *p* <0.001) [19]. Similarly, a record-linkage study in the United Kingdom found an RR of 9.4 (*p* <0.001) in SLE patients [29]. The above studies did not consider confounding factors such as medication, but the results are generally in agreement with our analysis.

Current literature suggests that glucocorticoids can suppress cellular immune responses through a variety of mechanisms, including inhibition of lymphokine effect and monocyte chemotaxis, depression of IL-1 and TNF production, and impairment of T cell activation [30]. Some case–control and cohort studies conducted in regions with low to medium burden of TB found a higher risk for TB reactivation in patients receiving corticosteroids (OR/RR of 1.7-4.9) [12–14]. Another study from Canada found that the risk of TB reactivation was only significantly increased when taking medium to high doses (≥10mg per day) of glucocorticoids [16]. A retrospective study from China showed that the accumulated doses of glucocorticoid (GC) (OR = 2.32, 95% CI 1.69–3.20, *P* < 0.001) were associated with TB [20]. Consistent with previous findings, history of long-term and high-dose steroid therapy (≥30mg/d and >4 weeks) was an independent risk factor for ATB in our study, suggesting a similar condition in regions with high burden of TB.

Our study showed that exposure to AZA within the past two years was a risk factor of borderline significant for ATB (OR = 2.095, *p* =0.054). AZA was found to significantly increase the risk of developing TB in patients with inflammatory bowel disease (RR = 6.27, *p* =0.046) [31]. A retrospective cohort study showed that the use of AZA was an independent risk factor for ATB after lung transplantation (OR = 10.6, *p* =0.038) [32]. Few studies evaluated the risk of ATB in patients with rheumatic diseases under treatment of AZA, a systematic review of randomized controlled trials found that the risk of ATB reactivation was higher when anti-TNF agents were combined with AZA as compared with anti-TNF monotherapy (OR = 13.3, *p* <0.001) [33]. Our study provides more evidence on this issue in patients with rheumatic diseases.

This study also found a significant association between ATB and previous TB infection (OR = 6.185, *p* <0.001). With the advent of molecular epidemiology, more and more evidence from genotyping studies suggest that recurrent TB is mainly caused by endogenous reactivation [34–36]. In our study, it was not possible to identify whether the recurrence of ATB was due to reactivation of latent infection or exogenous reinfection, but undoubtedly more attention associating TB prevention should be paid to patients with previous infection as they are more vulnerable to secondary ATB.

There are some limitations in this study. Firstly, pathogenic evidence was not obtained from two thirds of ATB cases. The molecular techniques for MTB diagnosis had not been approved by the National Medical Products Administration during the study period, so the proportion of TB patients confirmed by microbiology might be underestimated. Although only a third of ATB patients were diagnosed microbiologically, we established a strict diagnostic algorithm, including clinical manifestations, laboratory test results and imaging findings. Most importantly, we followed up patients for 12 weeks to determine whether the diagnostic anti-TB treatment was effective. Moreover, the diagnosis of ATB was independently made by two physicians and a senior doctor was consulted when there was disagreement. These measures increased the accuracy of ATB diagnosis. Secondly, the range of confidence interval was quite wide in certain types of rheumatic diseases due to the small number of patients. Although the point estimates are higher than those of the general population, it needs to be confirmed by further studies in a specific population. Thirdly, several immunosuppressant variables with few observations in ATB group may make the results less robust, it also needs to be confirmed by future studies with large-scale ATB patients using immunosuppressants.

In conclusion, the prevalence of ATB among patients with rheumatic diseases is higher than that of the general population in China. The risk factors including having SLE or BD, taking AZA within the past two years, exposing to long-term medium to high dosage of glucocorticoids and having evidence of previous TB should be considered in the individualized clinical assessment of the risk of ATB in patients with rheumatic diseases not taking anti-TNF medications.

**List of abbreviations**
ATB: active tuberculosis
AS: ankylosing spondylitis
AZA: azathioprine
BD: behcet’s disease
CI: confidence interval
CsA: cyclosporine A.
CTX: cyclophosphamide
DM: dermatomyositis
EGPA: eosinophilic granulomatosis with polyangiitis
FK506: tacrolimus
GCA: giant cell arteritis
GCs: glucocorticoids
GPA: granulomatosis with polyangiitis
IQR: interquartile range
LEF: leflunomide
MCTD: mixed connective tissue diseases
MMF: mycophenolate mofetil
MPA: microscopic polyangiitis
MTX: methotrexate
PAN: polyarteritis nodosa
PM: polymyositis
PsA: psoriatic arthritis
RA: rheumatoid arthritis
SASP: sulfasalazine
SD: standard deviation
SLE: systemic lupus erythematosus
SpA: spondyloarthropathy
SS: sjogren’s syndrome
SSc: systemic sclerosis
TA: takayasu's arteritis
TwHF: tripterygium wilfordii hook f

## Supplementary Material

Diagnostic_Criteria_for_the_Rheumatic_Disease.docx

Supplementary_material.docx

## Data Availability

The data that support the findings of this study are available from the corresponding author, Prof. Xiaoqing Liu, upon reasonable request.

## Geolocation information

China
