# Supplementary material for: Prevalence and risk factors of active tuberculosis in patients with rheumatic diseases: a multi-center, cross-sectional study in China
Source: Emerg Microbes Infect. 2021 Dec 6;10(1):2303–12. doi: 10.1080/22221751.2021.2004864 (PMC8654396; doi:10.1080/22221751.2021.2004864)
Supplement: Diagnostic_Criteria_for_the_Rheumatic_Disease.docx [file TEMI_A_2004864_SM8281.docx]

Diagnostic Criteria for the Rheumatic Disease

Systemic lupus erythematosus (SLE),[1] Rheumatoid arthritis (RA),[2] Sjogren's syndrome (SS),[3, 4] Systemic sclerosis (SSc),[5, 6] Mixed connective tissue disease (MCTD),[7] Polymyositis (PM),[8] Takayasu arthritis (TA),[9] Giant cell arthritis (GCA),[10] Polyarteritis nodosa (PAN),[11] Granulomatosis with polyangiitis (GPA),[11] Microscopic polyarteritis (MPA),[11] Eosinophilic granulomatosis with polyangiitis (EGPA),[11] Behcet’s disease (BD),[12, 13] Ankylosing spondylitis (AS)[14] and Psoriatic arthritis (PsA).[14]

1. Petri M, Orbai AM, Alarcon GS, et al. Derivation and validation of the Systemic Lupus International Collaborating Clinics classification criteria for systemic lupus erythematosus. *Arthritis Rheum* 2012; 64: 2677-2686.

2. Aletaha D, Neogi T, Silman AJ, et al. 2010 Rheumatoid arthritis classification criteria: an American College of Rheumatology/European League Against Rheumatism collaborative initiative. *Arthritis Rheum* 2010; 62: 2569-2581.

3. Vitali C, Bombardieri S, Jonsson R, et al. Classification criteria for Sjogren's syndrome: a revised version of the European criteria proposed by the American-European Consensus Group. *Annals of the rheumatic diseases* 2002; 61: 554-558.

4. Shiboski SC, Shiboski CH, Criswell L, et al. American College of Rheumatology classification criteria for Sjogren's syndrome: a data-driven, expert consensus approach in the Sjogren's International Collaborative Clinical Alliance cohort. *Arthritis Care Res (Hoboken)* 2012; 64: 475-487.

5. Preliminary criteria for the classification of systemic sclerosis (scleroderma). Subcommittee for scleroderma criteria of the American Rheumatism Association Diagnostic and Therapeutic Criteria Committee. *Arthritis Rheum* 1980; 23: 581-590.

6. van den Hoogen F, Khanna D, Fransen J, et al. 2013 classification criteria for systemic sclerosis: an American college of rheumatology/European league against rheumatism collaborative initiative. *Annals of the rheumatic diseases* 2013; 72: 1747-1755.

7. Sharp GC. Diagnostic criteria for classifcation of MCTD. *Mixed Connective Tissue Disease and Ami‐Nuclear Antibodies* 1987.

8. Bohan A, Peter JB. Polymyositis and dermatomyositis (first of two parts). *N Engl J Med* 1975; 292: 344-347.

9. Arend WP, Michel BA, Bloch DA, et al. The American College of Rheumatology 1990 criteria for the classification of Takayasu arteritis. *Arthritis Rheum* 1990; 33: 1129-1134.

10. Hunder GG, Bloch DA, Michel BA, et al. The American College of Rheumatology 1990 criteria for the classification of giant cell arteritis. *Arthritis Rheum* 1990; 33: 1122-1128.

11. Jennette JC, Falk RJ, Andrassy K, et al. Nomenclature of systemic vasculitides. Proposal of an international consensus conference. *Arthritis Rheum* 1994; 37: 187-192.

12. Criteria for diagnosis of Behcet's disease. International Study Group for Behcet's Disease. *Lancet* 1990; 335: 1078-1080.

13. International Team for the Revision of the International Criteria for Behcet's D. The International Criteria for Behcet's Disease (ICBD): a collaborative study of 27 countries on the sensitivity and specificity of the new criteria. *J Eur Acad Dermatol Venereol* 2014; 28: 338-347.

14. Dougados M, van der Linden S, Juhlin R, et al. The European Spondylarthropathy Study Group preliminary criteria for the classification of spondylarthropathy. *Arthritis Rheum* 1991; 34: 1218-1227.
