# Supplementary material for: Prevalence and risk factors of active tuberculosis in patients with rheumatic diseases: a multi-center, cross-sectional study in China
Source: Emerg Microbes Infect. 2021 Dec 6;10(1):2303–12. doi: 10.1080/22221751.2021.2004864 (PMC8654396; doi:10.1080/22221751.2021.2004864)
Supplement: Supplementary_material.docx [file TEMI_A_2004864_SM8280.docx]

In the multivariable logistic regression model, the Chi-Square of Hosmer and Lemeshow Goodness-of-Fit was 1.867 and its P value was 0.760, the AUROC was 0.733(95CI%: 0.675-0.791). As for the Collinearity Statistics, all tolerance were around 0.9 and VIF around 1. No interaction between the variables in the model were found (P values > 0.05).

| **Coefficients^a^** | | | | | | | | |
| --- | --- | --- | --- | --- | --- | --- | --- | --- |
| Model |  | Unstandardized Coefficients | | Standardized Coefficients |  |  | Collinearity Statistic | |
|  |  | B | Std. Error | Beta | t | Sig. | Tolerance | VIF |
| 1 | (Constant) | -.031 | .006 |  | -5.169 | .000 |  |  |
|  | age | .000 | .000 | -.019 | -1.967 | .049 | .934 | 1.071 |
|  | With evidence of previous TB | .032 | .004 | .068 | 7.332 | .000 | .997 | 1.003 |
|  | Duration of GCs≥30mg/d within past two years (weeks) | .009 | .002 | .037 | 3.665 | .000 | .840 | 1.190 |
|  | Type of rheumatic diseases | .000 | .001 | -.005 | -.485 | .627 | .842 | 1.188 |
|  | Use of CTX within past two years | .001 | .002 | .005 | .465 | .642 | .877 | 1.140 |
|  | Use of MMF within past two years | .003 | .003 | .009 | .881 | .378 | .910 | 1.099 |
|  | Use of AZA within past two years | .011 | .004 | .023 | 2.514 | .012 | .978 | 1.022 |
|  | Use of LEF within past two years | 8.750E-5 | .002 | .000 | .042 | .966 | .863 | 1.158 |
| 1. Dependent Variable: ATB or NOT | | | | | | | | |
